# Comparative evaluation of multimodal large language models for diagnostic accuracy in pediatric electrocardiography: a prospective comparative diagnostic accuracy study

**DOI:** 10.1007/s00431-026-06874-x

**Published:** 2026-03-24

**Authors:** Uğur Saraç, Ayşe Büşra Paydaş, Mustafa Gençeli, Talha Üstüntaş, Mehtap Yücel, Abdülkerim Çokbiçer, Fatih Şap, Tamer Baysal, Mehmet Burhan Oflaz

**Affiliations:** 1 Department of Pediatric Cardiology, Faculty of Medicine, Necmettin Erbakan University, Konya, Turkey; 2 Department of Pediatric Infectious Diseases, Faculty of Medicine, Necmettin Erbakan University, Konya, Turkey; 3 Department of Pediatrics, Faculty of Medicine, Necmettin Erbakan University, Konya, Turkey; 4 Department of Public Health, Faculty of Medicine, Necmettin Erbakan University, Konya, Turkey

**Keywords:** Artificial intelligence, Large language models, Pediatric electrocardiography, Diagnostic accuracy, Likelihood ratios, Arrhythmia detection

## Abstract

We evaluated three multimodal LLMs, ChatGPT (GPT-5.2), Gemini 3, and Microsoft Copilot, in pediatric ECG interpretation, focusing on clinically significant abnormalities and emergency arrhythmias with likelihood ratios as primary outcome measures. This prospective comparative diagnostic accuracy study (STARD/STARD-AI) included 264 pediatric patients with 12-lead ECGs (November 2024–November 2025). De-identified images were submitted via standardized zero-shot prompt. Three blinded pediatric cardiologists established the reference diagnosis by majority-vote consensus. Cases were classified as Tier 1 (normal), Tier 2 (abnormal, non-urgent), or Tier 3 (urgent). Two binary endpoints were assessed: clinically significant abnormality (Tier 2 + 3 vs Tier 1) and emergency abnormality (Tier 3 vs Tier 1 + 2). Clinically significant abnormalities were present in 54.5% of patients. AUC values ranged from 0.550 to 0.623, reflecting modest discrimination. For the clinically significant endpoint, + LR values were 2.05 (ChatGPT), 1.26 (Gemini), and 1.21 (Copilot); − LR values were 0.68, 0.55, and 0.81, indicating limited rule-in and insufficient rule-out utility. For the emergency endpoint, Gemini achieved 100% sensitivity (95% CI = 85.1–100.0) with − LR 0.07 (95% CI = 0.00–1.12) in a small subgroup (*n* = 22); however, specificity of 30.2% and + LR of 1.40 indicate overcalling rather than diagnostic precision. No model achieved clinically meaningful rule-in utility for either endpoint.

*Conclusions*: Current multimodal LLMs showed limited diagnostic utility in pediatric ECG interpretation, with + LR values near 1.0 across both endpoints. Standalone deployment is not supported; these tools may at most serve as adjunctive screening aids under clinician oversight.

**Table:** 

**What is Known:** • *Deep learning algorithms trained on large ECG datasets perform well in adult populations, but evidence in pediatric ECG interpretation is limited.* • *General-purpose LLMs show variable accuracy in medical examinations; reliability in subspecialty domains such as pediatric cardiology remains unproven.*	
**What is New:** • *This is the[FCA1] first head-to-head comparative diagnostic accuracy study of multimodal LLMs in pediatric ECG evaluation, using likelihood ratios as primary outcome measures.* • *All three LLMs showed limited rule-in utility (+LR near 1.0); Gemini achieved potentially meaningful rule-out performance for emergency arrhythmias (−LR = 0.07), but with wide confidence intervals reflecting the small emergency subgroup (n = 22).* • *Gemini’s 100% sensitivity in the emergency subgroup reflects overcalling (specificity 30.2%) consistent with a triage/screening behavior rather than diagnostic precision.*

**Supplementary Information:**

The online version contains supplementary material available at 10.1007/s00431-026-06874-x.

## Introduction

Electrocardiography (ECG) is a safe, inexpensive, and widely available diagnostic tool in pediatric cardiology. Artificial intelligence applications have expanded rapidly in image-based diagnostics including ECG interpretation [1]. Large language models (LLMs) such as ChatGPT (OpenAI), Copilot (Microsoft), and Gemini (Google) have attracted interest as clinical decision-support tools [2], though GPT-4 shows variable accuracy across medical domains [3] and inconsistent ECG analysis performance [4]. Existing evaluations are largely based on GPT-4; the release of GPT-5.2 with enhanced multimodal capabilities warranted validation in pediatric diagnostics.

Purpose-built deep learning models trained on large clinical datasets can now predict ventricular dysfunction and identify pathologies including aortic stenosis, hypertrophic cardiomyopathy, long QT syndrome, and atrial septal defect [5–8], and some extract features beyond routine clinical reading, such as signals associated with hyperkalemia, atrial fibrillation risk, and mortality [9, 10].

Pediatric ECG interpretation poses distinct challenges: age-dependent physiological variation, wide normative ranges, and the complexity of congenital heart disease [11]. Interobserver variability and reliance on subspecialty expertise can lead to delayed or missed diagnoses, and AI-assisted reading could improve diagnostic equity, though realizing that potential requires diverse datasets and multicenter collaboration [12].

The present study evaluated the diagnostic performance of three multimodal LLMs, ChatGPT (GPT-5.2), Gemini 3, and Microsoft Copilot, in pediatric ECG interpretation. The analysis focused on the detection of clinically significant ECG abnormalities and emergency arrhythmias, with positive and negative likelihood ratios (+ LR/− LR) as the primary clinically interpretable performance measures.

## Materials and methods

### Study design and participants

A prospective comparative diagnostic accuracy study was conducted at our pediatric clinic between November 2024 and November 2025, reported in accordance with the Standards for Reporting Diagnostic Accuracy Studies (STARD) and STARD-AI guidelines. Five hundred consecutive pediatric 12-lead ECG recordings archived during the study period were assessed for eligibility. Records with substantial artifacts affecting interpretation were excluded, as were cases for which clinical records required for reference standard adjudication were unavailable. The final analytic cohort comprised 264 patients under 18 years of age with complete clinical assessments and technically adequate 12-lead ECG recordings.

All ECGs were scanned using a Canon i-SENSYS MF6180dw scanner and digitized in JPEG format at 300 dpi to ensure consistent lighting and resolution. Patient identifiers and automated device interpretations were redacted before submission to the AI models.

Freely accessible versions of ChatGPT (GPT-5.2), Gemini 3 (Thinking mode), and Microsoft Copilot (Smart mode), as available in November 2025, were evaluated using a standardized zero-shot prompt (supplementary material). Models were instructed to interpret the ECG image, provide a single primary diagnosis, and specify whether the finding was clinically significant. Each ECG was evaluated in a single model run; temperature and sampling randomness parameters are not user-adjustable in the retail interfaces used, and default system settings were applied throughout. Within-model consistency across repeated runs was therefore not assessed. Responses were compared against the definitive reference standard diagnosis.

Three pediatric cardiologists, each with a minimum of 20 years of clinical experience, established the definitive diagnosis based on 12-lead ECG findings together with available clinical information. All three cardiologists were blinded to AI model outputs throughout the adjudication process. In cases of interpretive disagreement, the final diagnosis was determined by majority vote (2 of 3 cardiologists). Formal quantification of interobserver agreement prior to majority adjudication was not performed; this is acknowledged as a limitation.

### Outcome classification

Cases were classified into three tiers, defined a priori before model evaluation:

- **Tier 1 (Normal)**: Sinus arrhythmia, low atrial rhythm, early repolarization pattern, physiological right ventricular dominance, wandering atrial pacemaker, sinus bradycardia, and sinus tachycardia.
- **Tier 2 (Abnormal, non-urgent)**: All clinically significant ECG abnormalities not meeting Tier 3 criteria.
- **Tier 3 (Urgent**): Supraventricular tachycardia, ectopic atrial tachycardia, permanent junctional reciprocating tachycardia, left posterior fascicular ventricular tachycardia, ventricular tachycardia, and complete atrioventricular block.

Two prespecified binary endpoints were analyzed: (i) clinically significant abnormality, defined as Tier 2 + 3 versus Tier 1; and (ii) emergency abnormality, defined as Tier 3 versus Tier 1 + 2. Because the LLMs generated only a “clinically significant vs not significant” classification rather than a separate “urgent” output, the emergency endpoint was operationalized by applying model outputs to the Tier 3 vs (Tier 1 + 2) reference partition. False positives from the all-arrhythmias contingency table served as the true-negative reference group for emergency specificity calculations.

### Statistical analysis

Statistical analysis was performed in SPSS 18.0 (SPSS Inc., Chicago, IL, USA). Data normality was assessed through visual inspection of histograms and probability plots and the Kolmogorov–Smirnov test. Numerical data are expressed as median (1st–3rd quartile); categorical data as frequencies and percentages. Positive and negative likelihood ratios (+ LR and − LR), reported with 95% confidence intervals, served as the primary clinically interpretable performance measures. Calculations followed standard formulas: + LR = sensitivity/(1 − specificity) and − LR = (1 − sensitivity)/specificity. A continuity correction of 0.5 was applied to zero cells for Gemini in the emergency subgroup (FN = 0). Likelihood ratios are reported descriptively; no formal inferential testing was performed for between-model LR differences, as comparisons were framed in terms of confidence interval width and overlap. Secondary measures included sensitivity, specificity, positive predictive value (PPV), negative predictive value (NPV), accuracy, and F_1_ score, all reported with 95% confidence intervals. Discriminatory power for the clinically significant endpoint was additionally assessed by receiver operating characteristic (ROC) analysis, with the area under the curve (AUC), 95% CI, and *p*-value reported for each model. ROC analysis was not performed for the emergency endpoint because all model outputs for Tier 3 cases were classified as clinically significant, precluding distinction between positive and negative classes. Pairwise comparisons of sensitivity, specificity, and overall accuracy between models were performed using exact two-sided McNemar’s tests on discordant pairs. The Holm–Bonferroni correction was applied to the three pairwise comparisons within each outcome and metric, with adjusted *p*-values reported alongside raw values. Statistical significance was set at *p* < 0.05.

### Ethical considerations

The study protocol was approved by the Necmettin Erbakan University Faculty of Medicine Ethics Committee (approval no. 2025/6232). Written informed consent was obtained from parents or legal guardians of all participants prior to enrollment. All procedures were conducted in accordance with the Declaration of Helsinki and applicable local regulations governing human research.

## Results

A total of 264 patients were included, with a median age of 108.0 (IQR = 48.0–144.0) months; 43.6% (*n* = 115) were female and 56.4% (*n* = 149) were male. Normal sinus rhythm (15.5%), complete right bundle branch block (6.8%), T-wave inversion (6.8%), and premature ventricular contractions (6.1%) were the most frequently observed ECG findings. By three-tier classification, 120 patients (45.5%) had Tier 1 (normal) ECGs, 122 (46.2%) had Tier 2 (abnormal, non-urgent) findings, and 22 (8.3%) had Tier 3 (urgent) arrhythmias requiring emergency intervention. Clinically significant ECG abnormalities (Tier 2 + 3) were present in 144 patients (54.5%) (Table 1).

**Table 1:** Patient demographics and distribution of electrocardiographic diagnoses (*N* = 264)

Variables	Total cohort (*N* = 264)
Age (months), median (IQR)	108.0 (48.0–144.0)
**Sex, *n* (%)**	
Female	115 (43.6)
Male	149 (56.4)
**Electrocardiographic diagnosis, *n* (%)**	
Normal sinus rhythm	41 (15.5)
Complete right bundle branch block	18 (6.8)
T-wave inversion	18 (6.8)
Premature ventricular contractions	16 (6.1)
Incomplete right bundle branch block	14 (5.3)
Sinus arrhythmia	14 (5.3)
Wolff-Parkinson-White pattern	13 (4.9)
Premature atrial contractions	12 (4.5)
Artifact	11 (4.2)
First-degree atrioventricular block	10 (3.8)
Left ventricular hypertrophy	10 (3.8)
Physiological right ventricular dominance	8 (3.0)
Low atrial rhythm	8 (3.0)
Supraventricular tachycardia	8 (3.0)
Early repolarization pattern	7 (2.7)
Wandering atrial pacemaker	7 (2.7)
Superior QRS axis	6 (2.3)
Sinus tachycardia	5 (1.9)
Sinus bradycardia	5 (1.9)
Right ventricular hypertrophy	5 (1.9)
Junctional (nodal) rhythm	4 (1.5)
Ectopic atrial tachycardia	4 (1.5)
Complete (third-degree) atrioventricular block	4 (1.5)
Long QT syndrome	4 (1.5)
Left posterior fascicular ventricular tachycardia	3 (1.1)
Left bundle branch block	3 (1.1)
Ventricular tachycardia	2 (0.8)
Arrhythmogenic cardiomyopathy	2 (0.8)
Second-degree atrioventricular block (Mobitz Type I)	1 (0.4)
Permanent junctional reciprocating tachycardia	1 (0.4)
**Three-tier classification, *n* (%)**	
Tier 1: normal	120 (45.5)
Tier 2: abnormal, non-urgent	122 (46.2)
Tier 3: urgent	22 (8.3)

*IQR*, interquartile range. Three-tier classification: Tier 1 = normal ECG; Tier 2 = abnormal, non-urgent clinically significant abnormality; Tier 3 = urgent abnormality requiring emergency intervention

**Fig. 1:**
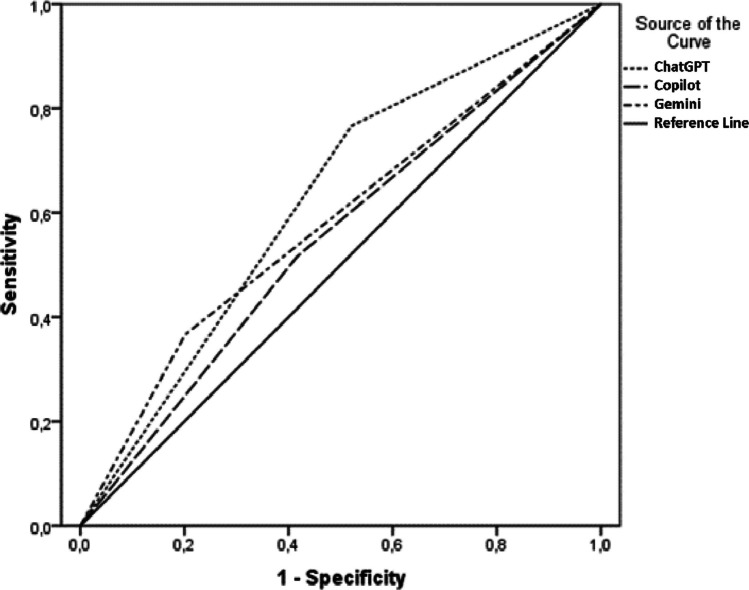
Receiver operating characteristic (ROC) curves for detection of clinically significant ECG abnormalities (Tier 2 + 3 vs Tier 1) by multimodal large language models. ChatGPT (GPT-5.2): AUC = 0.623 (95% CI = 0.555–0.690); Gemini 3: AUC = 0.583 (95% CI = 0.513–0.652); Copilot: AUC = 0.550 (95% CI = 0.480–0.620). The diagonal line represents the reference of no discrimination (AUC = 0.50). AUC values in the 0.55–0.62 range reflect modest discrimination that does not support standalone clinical deployment

AUC values for detecting clinically significant abnormalities (Tier 2 + 3 vs Tier 1) were 0.623 (95% CI = 0.555–0.690) for ChatGPT, 0.583 (0.513–0.652) for Gemini, and 0.550 (0.480–0.620) for Copilot (Fig. 1), indicating limited discrimination well below clinically adequate thresholds. ROC analysis was not performed for the emergency endpoint, as all Tier 3 model outputs were classified as clinically significant.

Table 2 (Section A) presents the full diagnostic performance metrics with 95% confidence intervals. The three models exhibited markedly different sensitivity–specificity trade-offs. Gemini achieved the highest sensitivity (79.9%; 95% CI = 72.6–85.6%) but the lowest specificity (36.7%; 95% CI = 28.6–45.6%). ChatGPT achieved the highest specificity (76.7%; 95% CI = 68.3–83.3%) but the lowest sensitivity (47.9%; 95% CI = 39.9–56.0%). Copilot showed intermediate performance on both dimensions (sensitivity 58.3%, specificity 51.7%).

**Table 2:** Diagnostic performance metrics of LLMs against the expert reference standard (95% confidence intervals)

Performance metric	ChatGPT	Copilot	Gemini 3
**Section A: All clinically significant abnormalities (Tier 2 + 3 vs Tier 1)**			
Correct classifications (*n*)	161	146	159
True positive (*n*)	69	84	115
False positive (*n*)	28	58	76
False negative (*n*)	75	60	29
True negative (*n*)	92	62	44
Sensitivity % (95% CI)	47.9 (39.9–56.0)	58.3 (50.2–66.1)	79.9 (72.6–85.6)
Specificity % (95% CI)	76.7 (68.3–83.3)	51.7 (42.8–60.4)	36.7 (28.6–45.6)
PPV % (95% CI)	71.1 (61.4–79.2)	59.2 (50.9–66.9)	60.2 (53.1–66.9)
NPV % (95% CI)	55.1 (47.5–62.4)	50.8 (42.1–59.5)	60.3 (48.8–70.7)
** + LR (95% CI)**	**2.05 (1.42–2.96)**	**1.21 (0.96–1.52)**	**1.26 (1.08–1.48)**
** − LR (95% CI)**	**0.68 (0.56–0.82)**	**0.81 (0.62–1.05)**	**0.55 (0.37–0.82)**
Accuracy % (95% CI)	61.0 (55.0–66.7)	55.3 (49.3–61.2)	60.2 (54.2–65.9)
F_1_ score	0.57	0.59	0.69
**Section B: Emergency arrhythmias (Tier 3 vs Tier 1 + 2)**			
True positive (*n*)	13	17	22
False positive (*n*)	84	125	169
False negative (*n*)	9	5	0
True negative (*n*)	158	117	73
Sensitivity % (95% CI)	59.1 (38.7–76.7)	77.3 (56.6–89.9)	100.0 (85.1–100.0)
Specificity % (95% CI)	65.3 (59.1–71.0)	48.3 (42.1–54.6)	30.2 (24.7–36.2)
PPV % (95% CI)	13.4 (8.0–21.6)	12.0 (7.6–18.3)	11.5 (7.7–16.8)
NPV % (95% CI)	94.6 (90.1–97.1)	95.9 (90.8–98.2)	100.0 (95.0–100.0)
** + LR (95% CI)**	**1.70 (1.15–2.51)**	**1.50 (1.16–1.94)**	**1.40 (1.27–1.55)**
** − LR (95% CI)**	**0.63 (0.38–1.04)**	**0.47 (0.22–1.03)**	0.07 (0.00–1.12)*
Accuracy % (95% CI)	64.8 (58.8–70.3)	50.8 (44.8–56.7)	36.0 (30.4–41.9)
F_1_ score	0.22	0.21	0.21

+ *LR*, positive likelihood ratio; − *LR*, negative likelihood ratio; *PPV*, positive predictive value; *NPV*, negative predictive value. Bold rows indicate likelihood ratios (primary outcome measures). Likelihood ratios are reported descriptively; no formal inferential test was applied to LR differences between models. *For Gemini in the emergency subgroup, FN = 0; a continuity correction of 0.5 was applied to FN and TN before computing − LR, resulting in a wide confidence interval. Accuracy is reported as a secondary metric; it is prevalence-sensitive and should not be used as the primary basis for clinical interpretation in this enriched cohort

Likelihood ratios reflect the clinical utility of each model more directly than accuracy measures. ChatGPT had the highest + LR at 2.05 (95% CI = 1.42–2.96), indicating modest rule-in utility, while Gemini and Copilot yielded + LR values of 1.26 (95% CI = 1.08–1.48) and 1.21 (95% CI = 0.96–1.52), respectively; the Copilot interval includes 1.0, indicating no reliable rule-in utility. For rule-out, Gemini achieved the lowest − LR at 0.55 (95% CI = 0.37–0.82), followed by ChatGPT at 0.68 (0.56–0.82) and Copilot at 0.81 (0.62–1.05). None of these values approaches the threshold commonly cited for clinically meaningful rule-out (approximately 0.1–0.2). Overall accuracy was comparable across models (ChatGPT 61.0%, Gemini 60.2%, Copilot 55.3%) but is of limited clinical interpretability given the enriched cohort prevalence. Gemini achieved the highest F_1_ score (0.69) compared with Copilot (0.59) and ChatGPT (0.57).

**Fig. 2:**
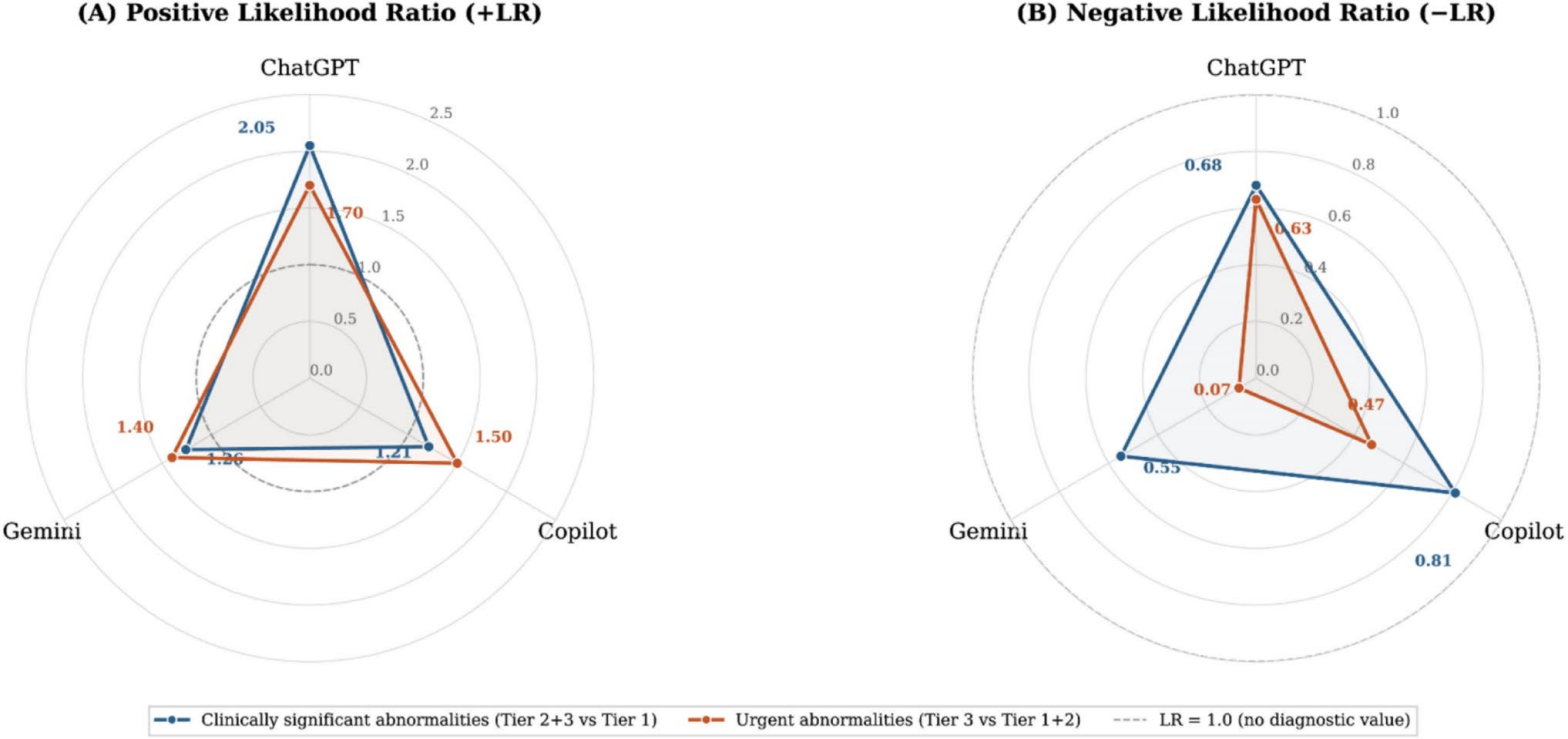
**A** Positive likelihood ratios (+ LR) for each multimodal LLM across two diagnostic endpoints: (i) clinically significant ECG abnormalities (Tier 2 + 3 vs Tier 1) and (ii) urgent arrhythmias requiring emergency intervention (Tier 3 vs Tier 1 + 2). Higher + LR values indicate stronger rule-in utility; a + LR of 1.0 indicates no change in post-test probability. Values close to 1.0, as observed across all models and both endpoints, indicate minimal diagnostic rule-in value. ChatGPT (GPT-5.2): + LR = 2.05 (clinically significant), 1.70 (emergency). Gemini 3: + LR = 1.26 (clinically significant), 1.40 (emergency). Copilot: + LR = 1.21 (clinically significant), 1.50 (emergency). **B** Negative likelihood ratios (− LR) for each multimodal LLM across the same two diagnostic endpoints. Lower − LR values indicate stronger rule-out utility; a − LR of 1.0 indicates no change in post-test probability. Values approaching 0.1 are generally considered clinically meaningful for rule-out. ChatGPT (GPT-5.2): − LR = 0.68 (clinically significant), 0.63 (emergency). Gemini 3: − LR = 0.55 (clinically significant), 0.07 (emergency; 95% CI = 0.00–1.12, computed with continuity correction). Copilot: − LR = 0.81 (clinically significant), 0.47 (emergency). Gemini’s emergency − LR of 0.07 should be interpreted with caution given the small subgroup size (*n* = 22) and wide confidence interval

Table 2 (Section B) presents the emergency endpoint analysis, with specificity calculated against the full non-emergency reference group (Tier 1 + 2, *n* = 242). Gemini classified all 22 urgent cases as clinically significant, achieving 100% sensitivity (95% CI = 85.1–100.0%), but at the cost of very low specificity (30.2%; 95% CI = 24.7–36.2%) and a + LR of only 1.40 (95% CI = 1.27–1.55), indicating negligible rule-in utility. Gemini’s − LR of 0.07 (95% CI = 0.00–1.12; computed with a 0.5 continuity correction for zero false negatives) represents the most favorable rule-out estimate among the three models; the wide confidence interval spanning 1.0, however, prevents a firm clinical conclusion. ChatGPT (sensitivity 59.1%; − LR 0.63) and Copilot (sensitivity 77.3%; − LR 0.47) did not achieve meaningful rule-out performance for emergency arrhythmias. No model produced a + LR indicative of useful rule-in utility for the emergency endpoint. The comparative distribution of the positive and negative likelihood ratios (+ LR and − LR) is summarized in Fig. 2A and B. PPV was low across all models (11.5–13.4%), consistent with the low emergency prevalence (8.3%) in this cohort.

Table 3 presents pairwise comparisons of accuracy, sensitivity, and specificity with the Holm–Bonferroni adjusted *p*-values. For clinically significant abnormalities, sensitivity differed significantly between ChatGPT and Gemini (*p* < 0.001) and between Gemini and Copilot (*p* < 0.001), while the ChatGPT–Copilot difference did not survive correction (*p* = 0.058). All pairwise specificity comparisons reached significance (ChatGPT vs Copilot: *p* < 0.001; ChatGPT vs Gemini: *p* < 0.001; Gemini vs Copilot: *p* = 0.006). In the emergency subgroup, sensitivity differences between models were non-significant after the Holm correction (all adjusted *p* ≥ 0.438), consistent with limited statistical power given the small subgroup size (*n* = 22). Specificity differed significantly between ChatGPT and Gemini (adjusted *p* = 0.029) and Gemini and Copilot (adjusted *p* < 0.001), so false-positive patterns diverged more than true-positive capture in this subgroup.

**Table 3:** Pairwise comparisons of accuracy, sensitivity, and specificity between LLMs: McNemar’s test with the Holm–Bonferroni correction

Metric/subgroup	Model A	Model B	A correct/B incorrect	A incorrect/B correct	*p* (raw)	*p* (adj, Holm)
**All clinically significant abnormalities**						
** Accuracy**						
	ChatGPT	Copilot	32	77	< 0.001	< 0.001
	ChatGPT	Gemini	15	109	< 0.001	< 0.001
	Gemini	Copilot	28	77	< 0.001	< 0.001
** Sensitivity**						
	ChatGPT	Copilot	20	35	0.058	0.058 (ns)
	ChatGPT	Gemini	10	56	< 0.001	< 0.001
	Gemini	Copilot	48	17	< 0.001	< 0.001
**Specificity**						
	ChatGPT	Copilot	42	12	< 0.001	< 0.001
	ChatGPT	Gemini	53	5	< 0.001	< 0.001
	Gemini	Copilot	11	29	0.006	0.006
**Emergency arrhythmias (Tier 3 vs Tier 1 + 2)**						
** Accuracy**						
	ChatGPT	Copilot	2	6	0.289	0.289 (ns)
	ChatGPT	Gemini	0	9	0.004	0.012
	Gemini	Copilot	0	5	0.063	0.125 (ns)
** Sensitivity**						
	ChatGPT	Copilot	6	3	0.508	0.750 (ns)
	ChatGPT	Gemini	1	4	0.375	0.750 (ns)
	Gemini	Copilot	9	3	0.146	0.438 (ns)
** Specificity**						
	ChatGPT	Copilot	35	21	0.081	0.081 (ns)
	ChatGPT	Gemini	6	19	0.015	0.029
	Gemini	Copilot	34	7	< 0.001	< 0.001

Discordant pairs reflect cases in which Model A and Model B produced different classifications (correct vs incorrect for accuracy; detected vs missed for sensitivity; correctly excluded vs falsely included for specificity). *p*-values from exact two-sided McNemar’s test. *p (adj)*, the Holm–Bonferroni adjusted *p*-value across three pairwise comparisons per metric per subgroup. *ns*, not significant after the Holm correction

## Discussion

Pediatric ECG interpretation by AI has received far less research attention than its adult counterpart [12]. Existing literature centers on deep learning models trained for single-disease detection in adults, targeting conditions such as atrial fibrillation, long QT syndrome, or left ventricular dysfunction [5–7]. Pediatric ECG assessment demands a fundamentally different approach: simultaneous differentiation of numerous rhythm and conduction disorders, accommodation of age-related physiological variation, and integration with clinical context that shifts as children grow.

Machine learning algorithms trained on large clinical datasets have achieved high accuracy in ECG classification [13]. Hannun et al. reported deep learning performance comparable to cardiologists in arrhythmia recognition, with high sensitivity across multiple rhythm classes [14]. That work, however, relied on a large, homogeneous adult dataset, limiting representation of the heterogeneous ECG findings encountered in pediatric practice. Ribeiro et al. showed that poor training data quality and insufficient dataset diversity degrade model performance [15], which reinforces that AI deployment in clinical settings requires caution, particularly where training data do not reflect the target population. Research examining general-purpose LLMs in pediatric ECG evaluation remains sparse. The present study is among the first to compare ChatGPT (GPT-5.2), Gemini 3, and Microsoft Copilot in detecting clinically significant pediatric ECG abnormalities within a comparative diagnostic accuracy framework using likelihood ratios as primary outcome measures.

Clinically significant ECG abnormalities were present in 54.5% of the 264 included patients, reflecting the enriched referral profile of a tertiary pediatric cardiology clinic. At this prevalence, accuracy becomes an unreliable metric: it is sensitive to class distribution and will appear deceptively high even for models with limited discriminatory value. Likelihood ratios were prioritized precisely because they assess clinical utility independent of cohort prevalence. Günay et al., using multiple-choice ECG questions, reported overall accuracy of 67.5% for ChatGPT-4o and 57.5% for Gemini, declining to 60% and 55% for more difficult ECGs [4]. Accuracy in the present study was comparable (ChatGPT 61.0%, Gemini 60.2%, Copilot 55.3%), but AUC values in the 0.55–0.62 range reflect only modest discrimination, well below thresholds required for standalone clinical deployment. Gritti et al. reported 54% accuracy for ChatGPT-4 on pediatric cardiology multiple-choice questions containing ECGs [16], consistent with the pattern observed here: LLMs show some capacity for ECG-related tasks but remain substantially limited in diagnostic precision. No comparable evaluation of Copilot was identified in the literature.

The models exhibited markedly different sensitivity–specificity profiles. Gemini achieved the highest sensitivity (79.9%) at the cost of low specificity (36.7%), minimizing missed cases but generating a high false-positive burden. ChatGPT showed a more conservative pattern, with the highest specificity (76.7%) but the lowest sensitivity (47.9%), reducing misclassification of normal findings at the expense of missed abnormalities. In principle, high-sensitivity models could serve first-pass screening roles while high-specificity models support confirmatory decisions. In practice, however, none achieved + LR values substantially above 2.0 or − LR values below 0.5 for the clinically significant endpoint, and neither profile meets the threshold for clinically reliable standalone performance.

Gemini’s classification of all 22 emergency arrhythmia cases as clinically significant, yielding 100% sensitivity, requires careful interpretation. The subgroup is small, and confidence intervals around all emergency endpoint estimates are correspondingly wide. The 100% sensitivity occurred alongside very low specificity (30.2%) and a + LR of only 1.40, a pattern consistent with overcalling rather than genuine diagnostic precision: Gemini classified nearly all inputs as potentially abnormal. The − LR of 0.07, while superficially suggestive of rule-out utility, carries a 95% confidence interval of 0.00–1.12 that includes 1.0, precluding a definitive clinical conclusion. What this pattern reflects is screening or triage behavior, not diagnostic accuracy, and it should not be taken as evidence of reliable emergency arrhythmia detection.

Because pathological and physiological cases are unevenly distributed across ECG categories in this cohort, accuracy alone remains misleading. Gemini’s F_1_ score of 0.69 indicates a stronger balance between sensitivity and positive predictive value than Copilot (0.59) or ChatGPT (0.57), suggesting somewhat improved reliability in capturing clinically significant abnormalities. The overarching conclusion from the likelihood ratio analysis, however, is unambiguous: positive LR values near 1.0 mean that a positive model output provides minimal incremental confirmation beyond prior probability, and no model achieved rule-out utility approaching clinically actionable thresholds for the all-abnormalities endpoint.

Rather than replacing specialist judgment, current general-purpose LLMs may at most serve an adjunctive triage function within clinical workflows, flagging cases that warrant specialist review in emergency departments and peripheral centers where pediatric cardiology expertise is limited. All model outputs require verification through clinical evaluation, and the risk of false positives generating unnecessary alarm or unwarranted investigations must be weighed against any potential screening benefit.

### Ethical and regulatory considerations

Deploying general-purpose LLMs in clinical diagnostic workflows raises ethical, safety, and regulatory considerations that extend beyond performance metrics. Medical-purpose use may trigger software-as-a-medical-device (SaMD) regulatory requirements depending on jurisdiction, requirements that these models are not currently designed to satisfy. LLMs occasionally produce confident but factually incorrect outputs (hallucinations), which in a clinical context could mislead clinicians or patients, particularly when outputs are accepted without critical appraisal. The opacity of LLM decision-making processes creates additional challenges for auditability and accountability. Such concerns reinforce that such tools, if used at all, should remain strictly adjunctive under active clinician oversight, with ultimate clinical and legal responsibility residing with the treating clinician and institution. Routine deployment outside validated, supervised frameworks is not supported by available evidence.

## Conclusions

Current multimodal LLMs demonstrated limited diagnostic utility in pediatric ECG interpretation. Positive likelihood ratios consistently near 1.0 indicate minimal rule-in value beyond pre-test probability across both endpoints. Only Gemini’s emergency − LR (0.07) approached a meaningful rule-out threshold, though the wide confidence interval (0.00–1.12) precludes a firm conclusion given the small subgroup (*n* = 22). Standalone clinical deployment is not supported. At most, high-sensitivity models may serve adjunctive, clinician-supervised triage in resource-limited settings, with the understanding that all outputs require verification and high false-positive rates must be anticipated. The models tested were trained predominantly on adult and text-based data; achieving meaningful pediatric ECG accuracy will require purpose-built, age-sensitive models trained on diverse pediatric datasets with multicenter validation.

### Strengths and limitations

Strengths include the prospective comparative design aligned with STARD/STARD-AI standards; a heterogeneous pediatric ECG spectrum; standardized de-identified image submission via a common zero-shot prompt; an a priori three-tier classification; likelihood ratios as primary outcome measures; and the Holm–Bonferroni correction for pairwise comparisons.

The study was conducted at a single tertiary clinic, and the patient population, acquisition conditions, and case distribution reflect our referral profile, limiting generalizability to primary care settings. Multicenter external validation is recommended. ECG images were standardized using a flatbed scanner (300 dpi), which does not reflect real-world acquisition via smartphone photography or screen captures, where glare, distortion, and variable resolution may affect performance. The emergency subgroup contained only 22 cases, with individual subtypes represented in very small numbers that preclude reliable subtype-specific estimates; these findings should be considered exploratory. Interobserver agreement among cardiologists was not formally quantified prior to majority adjudication. Pediatric ECG interpretation shows documented interobserver variability (kappa 0.44–0.66 for ventricular hypertrophy among experienced cardiologists [17]). Three independent readers with majority-vote adjudication and minimum 20 years’ experience reduce but do not eliminate the risk of systematic misclassification; future studies should document individual reads to permit reliability quantification. The zero-shot single-label prompt may have disadvantaged models in cases with multiple coexisting abnormalities, as clinical ECG interpretation typically involves multiple findings rather than a single forced label. Quantitative ECG parameters (PR interval, QRS duration, corrected QT, electrical axis) were not evaluated separately, representing an additional dimension of performance not captured here.

## Supplementary Information

Below is the link to the electronic supplementary material.

ESM 1 (DOCX 14.3 KB)

## Data Availability

The datasets generated and/or analyzed during the current study are available from the corresponding author on reasonable request.

## Abbreviations

AI: Artificial intelligence
ASD: Atrial septal defect
AUC: Area under the curve
AV: Atrioventricular
CHD: Congenital heart disease
CI: Confidence interval
dpi: Dots per inch
ECG: Electrocardiogram
IQR: Interquartile range
LLM: Large language model
LVH: Left ventricular hypertrophy
+ LR: Positive likelihood ratio
− LR: Negative likelihood ratio
NPV: Negative predictive value
PAC: Premature atrial contraction
PPV: Positive predictive value
PVC: Premature ventricular contraction
RBBB: Right bundle branch block
ROC: Receiver operating characteristic
RV: Right ventricular
SVT: Supraventricular tachycardia
WPW: Wolff-Parkinson-White
