# Supplementary material for: Comparative evaluation of multimodal large language models for diagnostic accuracy in pediatric electrocardiography: a prospective comparative diagnostic accuracy study
Source: Eur J Pediatr. 2026 Mar 24;185(4):206. doi: 10.1007/s00431-026-06874-x (PMC13009043; doi:10.1007/s00431-026-06874-x)
Supplement: Supplementary file 1 — (DOCX 14.3 KB) [file 431_2026_6874_MOESM1_ESM.docx]

The prompt used in the study is provided below.

“You are an experienced pediatric cardiologist.

I will show you a 12-lead pediatric ECG as an image.

Patient info:

- Age: AGE years

- Sex: SEX

Your task is to interpret ONLY the ECG and give a structured answer.

Very important:

- Use pediatric ECG norms (age-appropriate heart rate, axis, intervals, voltages).

- Do NOT assume any extra clinical information.

- If you are uncertain, choose the best option and note your uncertainty briefly.

Please follow this EXACT output format and do not add anything outside this template:

ECG INTERPRETATION

ECG description: 2–4 short sentences describing the main ECG findings

Main diagnosis: ONE main diagnosis sentence in clinical language

Clinically significant abnormality: YES or NO”
